# Respiratory symptoms and use of dust-control measures in New Zealand construction workers – A cross-sectional study

**DOI:** 10.1371/journal.pone.0266668

**Published:** 2022-04-07

**Authors:** Samuel Keer, Collin Brooks, Bill Glass, Dave McLean, Elizabeth Harding, Jeroen Douwes

**Affiliations:** Centre for Public Health Research, Massey University, Wellington, New Zealand; University of Melbourne, AUSTRALIA

## Abstract

Dust-exposed construction workers have an increased risk of respiratory symptoms, but the efficacy of dust-control measures remains unclear. This study compared respiratory symptoms, using a modified European Community Respiratory Health Survey questionnaire, between construction workers (n = 208) and a reference group of bus drivers and retail workers (n = 142). Within the construction workers, we assessed the effect of collective (on-tool vacuum/’wet-cut’ systems) and personal (respirators) exposure controls on symptom prevalence. Logistic regression assessed differences between groups, adjusted for age, ethnicity, and smoking status. Construction workers were more likely to cough with phlegm at least once a week (OR 2.4, 95% CI 1.2–4.7) and cough with phlegm ≥3 months/year for ≥2 years (OR 2.8, CI 1.2–7.0), but they had similar or fewer asthma symptoms. Construction workers who had worked for 11–20 years reported more cough/phlegm symptoms (OR 5.1, 1.7–15.0 for cough with phlegm ≥3 months/year for ≥2 years) than those who had worked <10 years (OR 1.9, 0.6–5.8), when compared to the reference group. Those who used ‘wet-cut’ methods reported less cough with phlegm, although the evidence for this association was weak (OR 0.4, CI 0.2–1.1 for cough with phlegm at least once a week); use of on-tool extraction showed a similar trend. No associations between respiratory protective equipment-use and symptoms were found. In conclusion, construction workers reported more symptoms suggestive of bronchitis, particularly those employed in the industry for >10 years. Use of collective dust exposure controls might protect against these symptoms, but this requires confirmation in a larger study.

## Introduction

Workers in the construction industry are regularly exposed to both organic (e.g. wood) and inorganic (e.g. silica) dust due to working in close proximity to processes involving cutting, drilling, sanding, grinding and breaking of wood, wood composites, concrete and masonry cement fibreboard [1–4]. Exposures may also occur due to disturbing deposited dust (e.g. cleaning). This, in addition to working in the confined spaces typical of many construction environments, may result in elevated and sustained exposures [4,5].

Previous studies have shown a higher prevalence of respiratory symptoms in dust-exposed construction workers, including wheezing and chronic cough [6]. In particular, cough with phlegm and symptoms suggestive of chronic bronchitis e.g. cough with phlegm daily for more than 3 months/year for more than 2 consecutive years) are commonly reported [6–10]. For example, in a study of 899 Danish construction workers [7], workplace dust exposure was associated with a higher prevalence of chronic cough and reduced lung function compared to an unexposed reference group, with those experiencing high daily dust exposures (such as demolition workers) particularly affected.

Few studies have been conducted in New Zealand, and these were either general population-based surveys with limited numbers of construction workers [11,12], or reported on asthma-related outcomes only [13]. Furthermore, these studies were conducted 10–20 years ago and workplace conditions are likely to have changed since then [14]. In particular, the efficacy and use of collective and personal dust exposure controls (e.g. local exhaust ventilation and RPE) are likely to have improved over the past decade [14–16], which (through the associated reduction in workplace exposures) may have resulted in a decline in respiratory symptoms [17]; however, relatively few studies of construction workers have directly assessed this [18].

In the current survey of construction workers and a reference group of retail workers and bus drivers [19,20] we assessed: 1) the prevalence of respiratory symptoms; 2) associations between employment duration and symptoms; and 3) whether use of measures to control dust is associated with a lower prevalence of respiratory symptoms.

## Methods

### Study population

Construction workers were recruited as described previously [21]. Briefly, after initial contact with large construction project management companies, we recruited 223 workers (aged 17–70 years) through 65+ subcontractors, representing a broad range of construction trades (e.g. general builders, scaffolders, carpenters, electricians, plumbers and painters) throughout the North Island of New Zealand. The comparison group (n = 281) comprised retail workers and bus drivers who were of similar socioeconomic status, and were recruited from similar geographical areas by approaching employers directly (retail workers) or through unions (bus drivers) [19]. All workers aged between 17 and 70 were invited to take part, and women were excluded from the analysis due to low numbers of female workers in the construction group. This project received ethical approval from the New Zealand Multi-Region Ethics Committee, Application MEC/10/08/081. Informed, written consent was obtained from all participants prior to participation in the study.

### Questionnaire

Were administered face-to-face. Construction workers were asked about the use of materials and tools which generate dust, participation in dusty work, and the use of collective and personal dust exposure control measures (based on a priori knowledge of on-site conditions, (obtained from exploratory site visits/surveys, and industry practice)). In particular, they were asked if they ever use ‘on-tool’ vacuum dust extraction (e.g. attachment of a portable vacuum cleaner to power tool exhaust ports) and/or ‘wet-cut’ methods (where water is continually fed onto the blade/bit of the tool). Use of respiratory protective equipment (RPE) (both ever/never and frequency) was also assessed. For questions relating to frequency of use of dust suppression techniques and RPE-use, responses were on a on a 5-point scale–‘seldom/never’, ‘sometimes’, ‘often’, ‘very often’ and ‘always’. Responses were dichotomised for subsequent analysis, with ‘seldom/never’ and ‘sometimes’ constituting a negative, and ‘often’, ‘very often’ and ‘always’ a positive response. Workers who reported no use of tools that generate dust were categorised as ‘not applicable’ for questions on both use of on-tool extraction and wet-cut methods.

The effect of work duration was assessed by dividing collision repair workers into tertiles which were then ‘rounded’ to the nearest decade of work duration, i.e. those who had worked in the industry <10 years, (average of 4.0 years; range 0.2–9.9), 10–20 years (average 14.3 years (10.1–19.9)), and >20 years (average 30.8 years (20.0–57.0)), as described previously [20,21]. Construction workers were also stratified according to their specific trade, based on company profiles and questionnaire responses i.e. Self-reported job title and work tasks performed.

Respiratory symptoms were assessed using questions based on the European Community Respiratory Health Survey (ECRHS) [22]. Self-reported asthma was identified using a well-characterised ECRHS definition involving a positive response to one or more of the following symptoms: 1) woken by shortness of breath in the last 12 months; 2) asthma attack in the last 12 months; or 3) current asthma medication. We also asked if asthma had been confirmed by a doctor. Self-reported symptoms associated with Chronic bronchitis were identified in accordance with the British Medical Research Council (BMRC) guidelines i.e. cough with phlegm almost daily for ≥3 months/year for ≥2 consecutive years [7,23]. Additional questions about respiratory symptoms (e.g. wheezing/whistling in the chest, breathlessness, chest tightness) and symptom frequency were also included. For questions relating to symptom frequency, responses were dichotomised as follows: ‘at least once a week’ and ‘at most twice a month’ (the latter including those who responded ‘Never/Seldom’).

Questions regarding potential confounders including age (years), ethnicity (Māori, Pacific or Other), and smoking status (never smoker, i.e. smoked fewer than 5 packets of cigarettes in whole life; ex-smoker, i.e. more than 5 packs in whole life and not a current smoker or; current smoker, i.e. more than 5 packs in whole life and still smoke [20,24]) were also included.

### Statistical analyses

All analyses were conducted using Stata version 13.1 (StataCorp LP, Texas, USA). Continuous and categorical data were analysed using t-test and chi-squared tests as appropriate. Prevalence odds ratios (pORs) comparing symptom prevalence between exposure groups were calculated using logistic regression. Comparisons were also made between the reference group and construction workers stratified according to both their employment duration and their specific trade. All analyses were adjusted for age (years), ethnicity (Māori, Pacific or Other), and smoking status.

## Results

Of the 223 construction workers who agreed to take part, 14 were excluded due to missing data. Women were also excluded (see above), leaving 208 construction and 142 reference workers (84 retail workers and 58 bus drivers) for inclusion in the analyses. Response rates were 64% and 34% for the construction and reference groups, respectively.

The characteristics of the construction workers (including numbers by trade) and reference groups are shown in Table 1. Construction workers were younger (mean 36.2 yrs. vs. 40.7 yrs., p<0.01), and a significantly higher proportion identified as Māori (the Indigenous population of New Zealand) compared to the reference group (32.2% vs. 9.2%, p<0.01). Smoking status was similar for both groups.

**Table 1:** Participant characteristics.

	**Reference workers (n = 142)**			**Construction workers (n = 208)**		
	**n**	**%**		**n**		**%**
**Ethnicity**						
Māori	13	9.2		67		32.2
Pacific	19	13.5		23		11.1
Other (incl. New Zealand European)	109	77.3		118		56.7
**Smoking Status**						
Non-smoker	60	42.3		86		41.4
Ex-smoker	34	23.9		45		21.6
Current smoker	48	33.8		77		37.0
	Mean	Range		Mean		Range
**Age**	40.7	17–70		36.2		17–64
**Duration of employment (Years)**	-	-		14.1		0.1–57
**Construction trade subcategories**						
**Job title**	**n**			**%**		
‘General builders’	83			39.9		
Scaffolders	36			17.3		
Carpenters	21			10.1		
Plumbers	20			9.6		
Managers/H&S staff	13			6.3		
Painters	12			5.8		
Electricians	11			5.3		
Groundworkers/plant operators	7			3.4		
Steel Fabricators	3			1.4		
Waterproofers	2			1.0		
	**n(%)**					
**Exposure control use**	**No**		**Yes**		**N/A**	
On-tool extraction	62 (29.8)		95 (45.7)		51 (24.5)	
Wet-cut methods	64 (30.8)		91 (43.8)		53 (25.5)	
RPE	91 (43.8)		91 (43.8)		26 (12.5)	

‘RPE’ = Respiratory Protective Equipment.

Construction workers had higher rates of cough with phlegm, e.g. cough with phlegm almost daily for >3 months/year for >2 years (OR 2.8, 95% CI 1.2–7.0), but were less likely to have had a self-reported attack of asthma (OR 0.4, 0.4–1.0) in the past 12 months (Table 2). Workers with medium employment duration (11–20 yrs) were more likely to have cough with phlegm symptoms than workers with the longest duration (>20 yrs; e.g. almost daily for >3 months/year for >2 years, OR 5.1, 1.7–15.0, Table 2). Those with the longest employment duration also had the lowest rates of wheezing or whistling in the chest (OR 0.4, 0.2–0.9) compared to the reference group.

**Table 2:** Prevalence odds ratios (OR) of respiratory symptoms in all construction workers and stratified by employment duration tertiles compared to reference workers.

	Retail workers	All Construction workers		Construction workers—employment duration (Mean)					
				<10 yrs (4.0)		11–20 yrs (14.3)		>20 yrs (30.8)	
	n = 142	n = 208		n = 99		n = 49		n = 60	
Respiratory symptoms:	N (%)	N (%)	OR (95%CI)^†^	N (%)	OR (95%CI) ^†^	N (%)	OR (95%CI) ^†^	N (%)	OR (95%CI) ^†^
Wheezing/whistling in chest in past 12 months.	50 (35.2)	55 (26.4)	**0.6 (0.4–1.0)**	27 (27.3)	0.6 (0.3–1.1)	17 (34.7)	1.0 (0.5–2.0)	11 (18.3)	**0.4 (0.2–0.9)**
Woken by shortness of breath in past 12 months	10 (7.0)	10 (4.8)	0.5 (0.2–1.3)	8 (8.1)	0.7 (0.3–2.2)	(0 (0.0)	-	2 (3.3)	0.4 (0.1–2.1)
Attack of asthma in the past 12 months	11 (7.8)	8 (3.9)	**0.4 (0.1–1.0)**	4 (4.0)	0.3 (0.1–1.2)	2 (4.1)	0.4 (0.1–2.0)	2 (3.3)	0.4 (0.1–1.9)
Asthma diagnosis	32 (22.5)	49 (23.6)	0.9 (0.5–1.6)	31 (31.3)	1.1 (0.6–2.2)	13 (26.5)	1.1 (0.5–2.4)	5 (8.3)	0.4 (0.1–1.1)
On medication for asthma	11 (7.8)	17 (8.2)	0.9 (0.4–2.1)	11 (11.1)	1.4 (0.5–3.7)	4 (8.2)	1.0 (0.3–3.3)	2 (3.3)	0.3 (0.1–1.7)
ECRHS asthma definition	17 (12.0)	22 (10.6)	0.7 (0.4–1.5)	13 (13.1)	0.9 (0.4–2.2)	6 (12.2)	0.9 (0.3–2.6)	3 (5.0)	0.3 (0.1–1.2)
Cough almost daily for at least part of the year	32 (22.5)	44 (21.5)	1.0 (0.5–1.7)	15 (15.5)	0.7 (0.3–1.4)	14 (24.6)	1.6 (0.7–3.6)	15 (25.4)	1.1 (0.5–2.4)
Dry cough at least once a week (vs. at most twice a month)	21 (14.8)	35 (16.9)	1.2 (0.7–2.3)	14 (14.1)	1.1 (0.5–2.4)	12 (24.5)	2.2 (0.9–2.1)	9 (15.3)	0.9 (0.4–2.3)
Cough almost daily for >3 months/yr for >2 years	21 (14.8)	36 (17.3)	1.3 (0.7–2.5)	11 (11.1)	0.9 (0.3–2.3)	12 (24.5)	1.5 (0.6–3.4)	13 (21.7)	1.5 (0.6–3.3)
Cough with phlegm almost daily for at least part of the year	15 (10.6)	38 (18.3)	1.9 (1.0–3.8)	12 (12.2)	1.1 (0.4–2.6)	13 (26.5)	**3.3 (1.4–8.1)**	13 (21.7)	2.4 (1.0–6.0)
Cough with phlegm at least once a week (vs. at most twice a month)	15 (10.6)	41 (19.7)	**2.4 (1.2–4.7)**	16 (16.2)	2.2 (0.9–5.1)	12 (24.5)	**3.4 (1.4–8.4)**	13 (21.7)	2.1 (0.9–5.0)
Cough with phlegm almost daily for >3 months/yr for >2 years	7 (4.9)	27 (13.0)	**2.8 (1.2–7.0)**	9 (9.1)	1.9 (0.6–5.8)	10 (20.4)	**5.1 (1.7–15.0)**	8 (13.3)	2.7 (0.8–8.5)

‘ECRHS’ = European Community Respiratory Health Survey.

ORs/CIs in bold indicate p values <0.05.

^†^ Adjusted for age, ethnicity and smoking status.

“-”No ORs available due to non-convergence in model.

Stratified analyses by specific construction trade among construction workers showed that ground workers/plant operators (n = 7, Table 3) had a higher prevalence of cough with phlegm symptoms compared to the reference workers, followed by painters (n = 12) and scaffolders (n = 36).

**Table 3:** Prevalence odds ratios of respiratory symptoms in individual construction trades compared to reference workers (n = 142).

	‘General Builder’	Scaffolder	Carpenter	Plumber	Manager/H&S	Painter	Electrician	Groundworker/ plant operator	Steel fabricator	Water proofer
	n (%)	n (%)	n (%)	n (%)	n (%)	n (%)	n (%)	n (%)	n (%)	n (%)
	83 (39.9)	36 (17.3)	21 (10.1)	20 (9.6)	13 (6.3)	12 (5.8)	11 (5.3)	7 (3.4)	3 (1.4)	2 (1.0)
	OR (95% CI) ^†^	OR (95% CI) ^†^	OR (95% CI) ^†^	OR (95% CI) ^†^	OR (95% CI) ^†^	OR (95% CI) ^†^	OR (95% CI) ^†^	OR (95% CI) ^†^	OR (95% CI)^†^	OR (95% CI) ^†^
**Respiratory symptoms:**										
Wheezing/whistling in chest in past 12 mnths.	0.6 (0.3–1.1)	0.9 (0.4–2.0)	0.4 (0.1–1.4)	0.4 (0.1–1.2)	0.7 (0.2–2.6)	0.7 (0.2–2.6)	0.4 (0.1–2.2)	1.7 (0.3–8.6)	-	-
Woken by shortness of breath in past 12 mnths	0.5 (0.2–1.9)	1.1 (0.3–4.9)	-	-	1.2 (0.1–10.8)	0.8 (0.1–7.4)	-	-	-	-
Attack of asthma in the past 12 months	0.4 (0.1–1.4)	1.2 (0.3–5.2)	-	-						
Asthma diagnosis	1.5 (0.8–2.8)	0.7 (0.3–1.9)	0.3 (0.1–1.4)	0.3 (0.1–1.5)	1.6 (0.4–6.0)	0.6 (0.1–3.1)	0.2 (0.0–2.1)	1.1 (0.1–10.8)	-	-
On medication for asthma	0.7 (0.2–2.1)	1.5 (0.4–5.5)	0.5 (0.1–4.3)	-	2.0 (0.4–10.4)	1.0 (0.1–9.2)	1.0 (0.1–8.4)	2.3 (0.2–23.3)	-	12.6 (0.6–247.3)
ECRHS asthma definition	0.7 (0.3–1.8)	1.5 (0.5–4.7)	0.3 (0.0–2.7)	-	1.4 (0.3–6.9)	0.5 (0.1–4.2)	0.7 (0.1–5.7)	1.7 (0.2–17.3)	-	4.0 (0.2–72.7)
Cough daily for at least part of the year	0.8 (0.4–1.7)	0.7 (0.2–2.1)	0.9 (0.3–3.2)	0.8 (0.2–2.6)	0.9 (0.2–5.0)	3.3 (0.9–12.6)	1.1 (0.2–6.2)	3.8 (0.7–21.1)	-	1.4 (0.1–24.6)
Dry cough at least once a week (vs. at most twice a month)	1.3 (0.6–2.7)	1.4 (0.5–4.4)	0.3 (0.0–2.5)	1.3 (0.4–4.4)	1.4 (0.3–7.2)	0.9 (0.2–4.3)	1.6 (0.3–8.4)	**5.9 (1.1–31.3)**	-	3.7 (0.2–65.5)
Cough almost daily for >3 months/yr for >2 years	0.9 (0.4–2.1)	1.2 (0.4–4.0)	1.6 (0.5–5.9)	0.7 (0.2–3.0)	1.6 (0.3–8.5)	2.7 (0.7–10.2)	2.0 (0.3–11.4)	**6.2 (1.1–35.0)**	-	2.9 (0.2–52.0)
Cough with phlegm daily for at least part of the year	1.6 (0.7–3.8)	**3.0 (1.0–9.1)**	1.6 (0.4–6.7)	1.1 (0.3–4.3)	0.9 (0.1–8.3)	**4.4 (1.2–17.0)**	-	**8.5 (1.5–48.2)**	3.2 (0.2–41.5)	3.9 (0.2–69.4)
Cough with phlegm at least once a week (vs. at most twice a month)	2.0 (0.9–4.5)	**3.7 (1.2–11.3)**	1.6 (0.4–6.3)	2.4 (0.7–8.0)	1.9 (0.4–10.1)	**4.5 (1.2–16.7)**	1.1 (0.1–10.1)	**6.1 (1.0–37.7)**	5.0 (0.4–69.8)	5.2 (0.3–93.5)
Cough with phlegm almost daily for >3 months/yr for >2 years	1.7 (0.6–5.2)	2.9 (0.7–12.4)	2.2 (0.4–11.9)	2.4 (0.5–10.8)	2.0 (0.2–18.6)	**9.5 (2.2–40.3)**	-	**25.4 (3.9–167.5)**	7.9 (0.5–129.5)	7.1 (0.4–136.0)

‘ECRHS’ = European Community Respiratory Health Survey.

ORs/CIs in bold indicate p values <0.05.

^†^ Adjusted for age, ethnicity and smoking status.

“-”No ORs available due to non-convergence in model.

A smaller proportion of construction workers who indicated using any on-tool extraction or wet-cut methods reported cough with phlegm symptoms compared to those who did not (e.g. cough with phlegm almost daily for >3 months/year for >2 years, OR 0.4, 95% CI 0.1–1.1, cough with phlegm, OR 0.4, 0.2–1.1, and dry cough, at least once a week vs. at most twice a month OR 0.4, 0.2–1.0, Table 4). No clear trends were observed between frequency of RPE use and symptoms (S3 Table).

**Table 4:** Prevalence odds ratios for use of on-tool vacuum extraction and wet-cut systems in construction workers.

	No to both on-tool extraction and wet-cut methods (n = 46, REF)	Yes to either (n = 125)		Not Applicable (n = 37) ^‡^	
	N/%	N/%	OR (95%CI) ^†^	N/%	OR (95%CI) ^†^
Wheezing/whistling in chest in past 12 months.	9 (19.6)	33 (26.4)	1.5 (0.6–3.7)	13 (35.1)	2.8 (1.0–7.9)
Woken by shortness of breath in past 12 months	3 (6.5)	5 (4.0)	0.6 (0.1–2.8)	2 (5.4)	0.8 (0.1–5.5)
Attack of asthma in the past 12 months	0 (0.0)	4 (3.2)	-	4 (10.8)	-
Asthma diagnosis	9 (19.6)	31 (24.8)	1.4 (0.5–3.3)	9 (24.3)	1.5 (0.5–4.7)
On medication for Asthma	2 (4.4)	11 (8.8)	2.4 (0.5–11.9)	4 (10.8)	2.9 (0.5–17.6)
ECRHS asthma definition	3 (6.5)	14 (11.3)	1.9 (0.5–7.3)	5 (13.5)	2.4 (0.5–11.4)
Cough daily for at least part of the year	8 (17.8)	26 (21.1)	0.9 (0.3–2.4)	10. (27.0)	2.3 (0.7–7.8)
Dry cough at least once a week (vs. at most twice a month)	10 (22.2)	16 (12.8)	0.4 (0.2–1.0)	9 (24.3)	1.1 (0.4–3.4)
Cough almost daily for >3 months/yr for >2 years	8 (17.4)	19 (15.2)	0.5 (0.2–1.5)	9 (24.3)	2.1 (0.6–7.3)
Cough with phlegm almost daily for at least part of the year	10 (21.7)	22 (17.6)	0.6 (0.2–1.4)	6 (16.2)	0.8 (0.2–2.6)
Cough with phlegm at least once a week (vs. at most twice a month)	11 (23.9)	23 (18.4)	0.4 (0.2–1.1)	7 (18.9)	0.6 (0.2–2.1)
Cough with phlegm almost daily for >3 months/yr for >2 years	8 (17.4)	14 (11.2)	0.4 (0.1–1.1)	5 (13.5)	0.8 (0.2–3.0)

‘ECRHS’ = European Community Respiratory Health Survey.

^†^ Adjusted for age, ethnicity and smoking status.

^‡^ = Participant responded to both use on-tool extraction and wet-cut methods questions with ‘not applicable’ (e.g. job role does not require use of tools which generate dust).

“-”No ORs available due non-convergence in model.

## Discussion

In this study, construction workers reported more bronchitis-related symptoms (particularly cough/phlegm almost daily for >3 months/year for >2 years) than reference workers, with workers with medium duration of employment in the industry (11–20 years) most likely to report symptoms. However, rates of self-reported asthma-related outcomes were similar or reduced. Workers who used on-tool dust extraction or wet-cut methods reported fewer symptoms compared to those who indicated no/limited use, although the evidence for this association was weak. No clear trends were observed with frequency of RPE use.

The higher prevalence of self-reported cough/phlegm symptoms is consistent with previous studies in construction workers [7,25]. A higher prevalence cough/phlegm symptoms but not those suggestive of asthma has also been reported previously; for example, in a large US population-based occupational cohort study [6], ‘Construction and associated trades’ workers were at significantly increased risk of ‘chronic bronchitis’ and ‘chronic cough’, but not asthma. Similarly, in an earlier general population study in New Zealand, work in the ‘construction and mining’ industry was associated with higher rates of chronic bronchitis [11], but not other respiratory symptoms often used to define asthma in occupational studies (e.g. wheeze [12]). The reasons for this are unclear, but we speculate that it may be that the majority of exposures in this setting in New Zealand are to irritants (e.g. dust) rather than classical asthma triggers such as allergens or other sensitisers [26–28], which may result in different symptomatology, whilst still being identified as “occupational asthma” (which as with “asthma” in general, encompasses a range of pathophysiologies and clinical manifestations) [29,30].

Although based on relatively small numbers in each work duration category, construction workers with a medium duration of employment reported more bronchitis-related symptoms compared to those with short and long employment duration (Table 2). Including age in the regression model did not affect the size of the effect estimates, but it did widen the confidence intervals somewhat. This indicates that there was some multicolinearity (see S4 Table for model excluding age); as a result, we cannot exclude the possibility that the association with employment duration is not, at least partially, driven by age. We found no clear work duration-response association. This may, at least in part, be due to the “healthy worker” effect—in which those susceptible to the effects of dust may leave high exposure roles or trades, leaving behind a group of less susceptible workers more able to tolerate such conditions [31]. Evidence of the healthy worker effect has also been reported in previous studies of workers exposed to dust, including several New Zealand-based studies [11,12].

Of the construction trades, groundworkers/plant operators had the highest prevalence of symptoms, followed by painters, scaffolders and plumbers (Table 3). Many of these workers (e.g. painters [32]) are known to be at risk of exposure to solvents, dust and other respiratory hazards (and associated respiratory effects) and others (e.g. groundworkers/plant operators, steel fabricators, electricians) are likely to regularly work, without wearing adequate RPE, in close proximity to dusty work and others who are using tools that generate dust (secondary exposures) [33–35]. However, analyses were based on small, in some cases very small numbers per strata, and findings should therefore be interpreted with caution. Sensitivity analyses excluding construction trades with fewer than 20 workers (managers/H&S staff, painters, electricians, groundworkers/plant operators, steel fabricators and waterproofers) resulted in weaker associations (S1 Table), but trends were comparable (Table 2). This suggests the higher prevalence of symptoms observed in construction workers as a whole is not solely attributable to these smaller groups, although we cannot rule out that unmeasured confounding may contribute to the differences between the two groups.

Several studies have shown that use of on-tool extraction and other local exhaust ventilation measures [34,35] and wet cut methods [4] are effective at reducing dust exposures, but to our knowledge our study is one of only a few to have directly assessed associations between use of dust exposure control measures and respiratory symptoms. In one Tanzanian study of 210 cement workers, a lower prevalence of respiratory symptoms (e.g. ‘chronic cough’ and ‘chronic sputum production’) and improved lung function was observed after the introduction of dust-control measures, which included installation of local area ventilation systems [18]. In our study any use of dust controls was associated with fewer symptoms related to dust exposure in construction workers (suggestive of bronchitis, e.g. cough with phlegm almost daily for >3 months/yr for >2 years, OR 0.4, 95% CI 0.1–1.1, Table 4) [6,11,12]. Associations were however weak, which may reflect the relatively small sample size and the limited data available on the control measures used, in addition to the subjective nature of the questionnaire data. Use of on-tool extraction and wet cut methods were combined into a single metric (as many reported using both), but similar trends were observed when analysed separately (S2 Table). Alternatively, dust control measures, as implemented in this industry, may not be fully effective, thus not contributing to measurable differences in symptom prevalence between those who use these measures and those who do not. Therefore, these results should be interpreted with caution. Nonetheless, given the importance of reducing exposures in this industry, we consider that further research to assess whether on-tool vacuum extraction and wet-cut methods may be protective against respiratory symptoms in these settings is warranted.

No clear trends were observed between frequency of RPE use and respiratory symptom rates (S3 Table). Previous studies in workers exposed to dust have shown that RPE may provide only limited protection against respiratory symptoms, often as a result of inconsistent use, improper fit and/or inappropriate selection of respirator type [36,37]. However, even when optimal, RPE programmes may only be partially effective (at least in the case of allergic asthma): Ilgaz, Moore [38] showed that workers with sensitiser-induced occupational asthma exposed to metal working fluid aerosols continued to experience falls (albeit of reduced magnitude) in peak expiratory flow after a strictly enforced RPE programme involving high quality air-fed respirators. The authors concluded that RPE cannot reliably replace controls at the source (e.g. LEV).

There were several limitations to this study. Respiratory health was assessed using self-reported symptoms, and therefore some misclassification may have occurred. However, the ECRHS has been used extensively to assess respiratory symptoms associated with occupational exposure, is well validated against clinical criteria [39–44], and standardised symptom assessment represents an important means of identifying populations “at risk” of effects associated with workplace exposures. The respiratory symptoms observed, particularly chronic cough/phlegm may also be caused by other disease processes, e.g. infection or Gastroesophageal Reflux Disease (GERD) [45]. However, we used the BMRC definition for chronic bronchitis (cough with phlegm almost daily for ≥3 months/year for ≥2 consecutive years), which is designed to minimise misclassification from these acute/more transient causes. Nonetheless, further studies including objective tests of lung function (spirometry) and inflammation (exhaled nitric oxide) and monitoring of workplace dust exposures would be useful to further clarify the nature and extent of any respiratory effects associated with exposures in this industry. The response rate in construction and reference workers was 64% and 34%, respectively. For the construction workers this is relatively high (for these types of surveys) and suggests that non-response bias, if present, would be small. The lower rate for the reference group may have resulted in some bias (i.e. those with respiratory symptoms may have been more likely to take part), but, if present, this would result in an underestimation (rather than over estimation) of the true effect. A number of the construction sub-trades were represented by only a very small number of workers, which limits our ability to draw valid conclusions from these data (re. trades most at risk of symptoms). However, we did observe a significantly higher prevalence of cough/phlegm symptoms in some subgroups, which may warrant further investigation in future studies involving larger sample sizes. Use of dust control measures and PPE was also self-reported, which may result in bias; for example, over-reporting of dust control equipment use through concern of admitting non-compliance with health and safety guidelines/regulations. However, if present, any bias would most likely lead to an underestimation of the true effect.

There were differences in age, ethnicity and smoking habits between construction workers and reference workers, but these were controlled for in the analyses. Duration of employment in the industry is generally not considered the most reliable proxy for cumulative exposure, but no historical data of dust exposure levels was available and exposure misclassification would likely lead to an underestimation of risk. We were also unable to assess current dust exposure levels, so it is unclear whether contemporary exposures contributed to the effects observed.

The questionnaire also focused primarily on the use of collective and personal measures to control dust exposures, but construction workers are also at risk of exposure to a variety of vapours, gases, and fumes, many of which may also cause respiratory symptoms (and may have contributed to the associations observed) [26–28]. It is likely that collective and personal exposure controls effective against dust would also be at least somewhat effective against other airborne contaminants [16], but we were unable to collect data on all potential exposures and specific control measures in this study. Finally, there could be unknown and unmeasured confounders that, at least in part, may explain some differences in symptom prevalence between the construction and reference groups.

Taken together, our findings suggest that despite the well-recognised risks of dust exposures and control options, elevated exposures and inadequate control measures may still be an issue in this industry (the effects observed in this study are unlikely to be solely attributed to historical exposures). Further studies are required to objectively assess contemporary dust exposure levels, their determinants, and the effectiveness of specific control measures. This will allow development of improved strategies to reduce dust exposure and resulting occupational disease in this industry.

In conclusion, construction workers exposed to dust continue to report bronchitis-related symptoms. Increased use of collective dust exposure controls may protect against these symptoms.

## Supporting information

S1 Table Prevalence odds ratios of respiratory symptoms in all construction workers/by employment duration tertiles compared to reference workers–Excluding trades represented by fewer than 20 workers (n = 48). (DOCX)

S2 Table Prevalence odds ratios for respiratory symptoms and use of dust suppression measures in construction workers (on-tool vacuum extraction and wet-cut systems). (DOCX)

S3 Table Prevalence odds ratios for respiratory symptoms and frequency of RPE use in construction workers (n = 182). (DOCX)

S4 Table Prevalence odds ratios of respiratory symptoms in construction workers stratified by employment duration tertiles compared to reference workers–Unadjusted for age. (DOCX)

S1 File (XLSX)
