# Supplementary material for: Respiratory symptoms and use of dust-control measures in New Zealand construction workers – A cross-sectional study
Source: PLoS One. 2022 Apr 7;17(4):e0266668. doi: 10.1371/journal.pone.0266668 (PMC8989237; doi:10.1371/journal.pone.0266668)
Supplement: S1 Table — (DOCX) [file pone.0266668.s001.docx]

Supplementary Table S1 –Prevalence odds ratios of respiratory symptoms in all construction workers and stratified by employment duration tertiles compared to reference workers – Excluding construction trades represented by fewer than 20 workers (n=48)

|  | **Retail workers** | **All Construction workers** | | **Construction workers - employment duration (Mean)** | | | | | |
| --- | --- | --- | --- | --- | --- | --- | --- | --- | --- |
|  |  |  |  | **<10 yrs (3.8)** | | **11-20 yrs (14.2)** | | **>20 yrs (31.7)** | |
|  | **n=142** | **n=160** | | **n=77** | | **n=42** | | **n=41** | |
|  | **N (%)** | **N (%)** | **OR (95%CI)** ^†^ | **N (%)** | **OR (95%CI)** ^†^ | **N (%)** | **OR (95%CI)** ^†^ | **N (%)** | **OR (95%CI)** ^†^ |
| Wheezing/whistling in chest in past 12 months. | 50 (35.2) | 41 (25.6) | **0.5 (0.3-0.9)** | 22 (28.6) | **0.6 (0.3-1.1)** | 12 (28.6) | 0.7 (0.3-1.5) | 7 (17.1) | **0.4 (0.1-0.9)** |
| Woken by shortness of breath in past 12 mnths | 10 (7.0) | 8 (5.3) | 0.4 (0.2-1.3) | 6 (7.8) | 0.6 (0.2-2.1) | 0 (0.0) | - | 2 (4.9) | 0.6 (0.1-3.7) |
| Attack of asthma in the past 12 mnths | 11 (7.8) | 7 (4.4) | **0.4 (0.1-1.1)** | 3 (3.9) | **0.3 (0.1-1.3)** | 2 (4.8) | 0.4 (0.1-2.2) | 2 (4.9) | 0.5 (0.1-2.8) |
| Asthma diagnosis | 32 (22.5) | 39 (24.4) | 0.9 (0.5-1.6) | 25 (32.5) | 1.1 (0.5-2.3) | 11 (26.2) | 1.0 (0.4-2.3) | 3 (7.3) | 0.3 (0.1-1.2) |
| On medication for asthma | 11 (7.8) | 11 (6.9) | 0.6 (0.2-1.7) | 7 (9.1) | 0.9 (0.3-2.8) | 2 (4.8) | 0.5 (0.1-2.4) | 2 (4.9) | 0.5 (0.1-2.5) |
| ECRHS asthma definition | 17 (12.0) | 16 (10.1) | 0.6 (0.3-1.4) | 9 (11.7) | 0.7 (0.3-1.9) | 4 (9.8) | 0.6 (0.2-2.1) | 3 (7.3) | 0.4 (0.1-1.8) |
| Cough almost daily for at least part of the year | 32 (22.5) | 29 (18.5) | 0.7 (0.4-1.4) | 11 (14.7) | 0.6 (0.2-1.3) | 10 (23.8) | 1.2 (0.5-2.8) | 8 (20.0) | 0.7 (0.3-1.9) |
| Dry cough at least once a week (vs. at most twice a month) | 21 (14.8) | 25 (15.7) | 1.1 (0.5-2.1) | 11 (14.3) | 1.0 (0.4-2.5) | 8 (19.1) | 1.5 (0.6-3.8) | 6 (15.0) | 0.8 (0.3-2.3) |
| Cough almost daily for >3 months/yr for >2 years | 21 (14.8) | 23 (14.4) | 0.9 (0.5-1.9) | 8 (10.4) | 0.7 (0.3-1.8) | 7 (16.7) | 1.2 (0.4-3.2) | 8 (19.5) | 1.1 (0.4-3.1) |
| Cough with phlegm almost daily for at least part of the year | 15 (10.6) | 27 (16.9) | 1.6 (0.8-3.3) | 7 (9.1) | 0.6 (0.2-1.7) | 10 (23.8) | **2.7 (1.1-7.1)** | 10 (24.4) | **3.0 (1.0-8.5)** |
| Cough with phlegm at least once a week (vs. at most twice a month) | 15 (10.6) | 29 (18.2) | **2.0 (1.0-4.0)** | 10 (13.0) | 1.5 (0.6-4.0) | 9 (21.4) | **2.5 (0.9-6.5)** | 10 (24.4) | 2.1 (0.8-5.5) |
| Cough with phlegm almost daily for >3 months/yr for >2 years | 7 (4.9) | 16 (10) | 1.8 (0.7-1.7) | 4 (5.2) | 0.7 (0.2-2.8) | 6 (14.3) | **2.8 (0.9-9.4)** | 6 (14.6) | **3.4 (0.9-13.8)** |

‘ECRHS’ = European Community Respiratory Health Survey

^†^ORs/CIs in bold indicate p values <0.05

Adjusted for age, ethnicity and smoking status

“-“ No ORs available due to non-convergence in model
