# Supplementary material for: Respiratory symptoms and use of dust-control measures in New Zealand construction workers – A cross-sectional study
Source: PLoS One. 2022 Apr 7;17(4):e0266668. doi: 10.1371/journal.pone.0266668 (PMC8989237; doi:10.1371/journal.pone.0266668)
Supplement: S2 Table — (DOCX) [file pone.0266668.s002.docx]

Supplementary table S2: Prevalence odds ratios for respiratory symptoms and use of dust suppression measures in construction workers (on-tool vacuum extraction and wet-cut systems).

|  | **On-tool vacuum extraction** | | | | | **Wet-cut methods** | | | | |
| --- | --- | --- | --- | --- | --- | --- | --- | --- | --- | --- |
|  | **No (n=62, REF)** | **Yes (n=95)** | | **N/A (n=51)** | | **No (n=64, REF)** | **Yes (n=91)** | | **N/A (n=53)** | |
|  | **N/%** | **N/%** | **OR (95%CI) ^†^** | **N/%** | **OR (95%CI) ^†^** | **N/%** | **N/%** | **OR (95%CI) ^†^** | **N/%** | OR (95%CI) ^†^ |
| Wheezing/whistling in chest in past 12 mnths. | 12 (19.4) | 28 (29.5) | 1.7 (0.8-3.9) | 28 (29.5) | 1.7 (0.8-3.9) | 14 (21.9) | 24 (26.4) | 1.4 (0.6-3.0) | 17 (32.1) | 1.8 (0.8-4.2) |
| Woken by shortness of breath in past 12 mnths | 3 (4.9) | 4 (4.2) | 0.8 (0.2-3.9) | 3 (5.9) | 1.2 (0.2-6.2) | 3 (4.7) | 5 (5.6) | 1.3 (0.3-6.1) | 2 (3.8) | 0.8 (0.1-4.9) |
| Attack of asthma in the past 12 mnths | 0 (0.0) | 4 (4.2) | **-** | 4 (7.8) | - | 0 (0.0) | 4 (4.2) | - | 4 (7.8) | - |
| Asthma diagnosis | 11 (17.7) | 25 (26.3) | 1.5 (0.7-3.6) | 13 (25.5) | 1.8 (0.7-4.7) | 11 (17.7) | 25 (26.3) | 1.2 (0.5-2.8) | 14 (26.4) | 1.2 (0.5-3.0) |
| On medication for asthma | 3 (3.2) | 10 (10.5) | 4.0 (0.8-20.0) | 5 (9.8) | 3.4 (0.6-19.2) | 3 (3.2) | 10 (10.5) | 2.8 (0.6-14.5) | 8 (15.1) | **5.9 (1.1-30.2)** |
| ECRHS asthma definition | 3 (4.9) | 12 (12.6) | 2.9 (0.7-11.2) | 7 (13.7) | 3.2 (0.8-13.4) | 3 (4.9) | 12 (12.6) | 1.7 (0.5-6.1) | 9 (17.0) | 3.2 (0.9-11.2) |
| Cough daily for at least part of the year | 11 (18.0) | 20 (21.3) | 0.9 (0.4-2.3) | 13 (26.0) | 1.9 (0.7-5.2) | 11 (18.0) | 20 (21.3) | 1.0 (0.4-2.4) | 13 (24.5) | 1.5 (0.6-4.1) |
| Dry cough at least once a week (vs. at most twice a month) | 12 (19.7) | 12 (12.6) | 0.5 (0.2-1.2) | 11 (21.6) | 1.1 (0.4-2.9) | 12 (19.7) | 12 (12.6) | **0.2 (0.1-0.6)** | 10 (18.9) | 0.6 (0.2-1.5) |
| Cough almost daily for >3 months/yr for >2 years | 11 (17.7) | 13 (13.7) | 0.5 (0.2-1.2) | 12 (23.5) | 1.7 (0.6-4.7) | 11 (17.7) | 13 (13.7) | 0.6 (0.2-1.5) | 11 (20.8) | 1.3 (0.5-3.5) |
| Cough with phlegm daily for at least part of the year | 15 (24.2) | 16 (16.8) | 0.5 (0.2-1.1) | 7 (13.7) | 0.5 (0.2-1.4) | 15 (24.2) | 16 (16.8) | 1.0 (0.4-2.3) | 8 (15.1) | 0.8 (0.3-2.3) |
| Cough with phlegm at least once a week (vs. at most twice a month) | 14 (22.6) | 20 (21.1) | 0.6 (0.2-1.3) | 7 (13.7) | 0.5 (0.2-1.3) | 14 (22.6) | 20 (21.1) | **0.4 (0.2-1.0)** | 10 (18.9) | 0.6 (0.2-1.4) |
| Cough with phlegm almost daily for >3 months/yr for >2 years | 11 (17.7) | 10 (10.5) | 0.4 (0.1-1.0) | 6 (11.8) | 0.6 (0.2-1.9) | 11 (17.7) | 10 (10.5) | 0.6 (0.2-1.6) | 7 (13.2) | 0.8 (0.3-2.5) |

‘ECRHS’ = European Community Respiratory Health Survey

^†^ORs/CIs in bold indicate p values <0.05

Adjusted for age, ethnicity and smoking status

“-“ No ORs available due to non-convergence in model
