# Supplementary material for: Respiratory symptoms and use of dust-control measures in New Zealand construction workers – A cross-sectional study
Source: PLoS One. 2022 Apr 7;17(4):e0266668. doi: 10.1371/journal.pone.0266668 (PMC8989237; doi:10.1371/journal.pone.0266668)
Supplement: S3 Table — (DOCX) [file pone.0266668.s003.docx]

Supplementary table S3 – Prevalence odds ratios for respiratory symptoms and frequency of RPE use in construction workers (n=182)

|  | **Respirator use** | | |
| --- | --- | --- | --- |
|  | **N/%** | |  |
|  | **Infrequent** | **Frequent** | **OR (95%CI) ˠ** |
|  | N (%) | N (%) |  |
|  | 119 | 63 |  |
| Wheezing/whistling in chest in past 12 mnths. | 26 (21.9) | 19 (13.2) | 1.5 (0.7-3.1) |
| Woken by shortness of breath in past 12 mnths | 5 (4.2) | 4 (6.4) | 1.6 (0.4-6.5) |
| Attack of asthma in the past 12 months | 4 (3.4) | 1 (1.6) | 0.3 (0.0-3.4) |
| Asthma diagnosis | 31 (26.1) | 12 (19.1) | 0.7 (0.3-1.6) |
| On medication for asthma | 8 (6.7) | 6 (9.5) | 1.6 (0.5-1.2) |
| ECRHS asthma definition | 11 (9.3) | 7 (11.1) | 1.2 (0.4-3.5) |
| Cough almost daily for at least part of the year | 22 (19.0) | 16 (26.4) | 1.0 (0.4-2.3) |
| Dry cough at least once a week (vs. at most twice a month) | 17 (14.4) | 9 (14.3) | 0.8 (0.3-2.1) |
| Cough almost daily for >3 months/yr for >2 years | 15 (12.6) | 14 (22.2) | 1.4 (0.6-3.6) |
| Cough with phlegm almost daily for at least part of the year | 19 (16.0) | 13 (20.6) | 1.1 (0.4-2.5) |
| Cough with phlegm at least once a week (vs. at most twice a month) | 22 (18.5) | 12 (19.1) | 0.8 (0.3-1.9) |
| Cough with phlegm almost daily for >3 months/yr for >2 years | 14 (11.8) | 9 (14.3) | 1.0 (0.4-2.7) |

‘ECRHS’ = European Community Respiratory Health Survey

^†^Adjusted for age, ethnicity and smoking status
